# Disparities, distribution, and determinants in appropriate timely initiation, number, and quality of antenatal care in Bangladesh: Evidence from Demographic and Health Survey 2017–18

**DOI:** 10.1371/journal.pgph.0002325

**Published:** 2023-08-23

**Authors:** Gulam Muhammed Al Kibria, Reese Crispen

**Affiliations:** 1 Department of International Health, Johns Hopkins Bloomberg School of Public Health, Baltimore, Maryland, United States of America; 2 Department of Epidemiology & Public Health, University of Maryland School of Medicine, Baltimore, Maryland, United States of America; Wachemo University, ETHIOPIA

## Abstract

Like many other low- and middle-income countries, Bangladesh experiences a disproportionately higher number of maternal and neonatal deaths compared to high-income countries. Despite this, a majority of pregnant women in Bangladesh do not receive appropriate antenatal care (ANC). We investigated the disparities, distribution, and determinants of the timing, number, and quality of ANC in this country. This cross-sectional study analyzed Bangladesh Demographic and Health Survey (BDHS) 2017–18 data on ever-married reproductive-age (i.e., 15-49-year-olds) women. After describing the study sample and proportions, multilevel logistic regression was applied to study determinants. The prevalence and odds of the studied outcomes were higher among women with higher parity, a higher education level, more highly educated husbands, urban residence, and residence in some administrative divisions (p<0.05). For instance, among women in the poorest, poorer, middle, richer, and richest wealth quintiles, the proportions of those who initiated ANC during the first trimester were 22.2% (95% confidence interval (CI): 19.6–25.0), 30.1% (95% CI: 27.1–33.2), 35.1% (95% CI: 31.7–38.6), 38.5% (95% CI: 35.2–42.0), and 61.0% (95% CI: 57.5–64.3). Then, compared to women in the poorest wealth quintile, the adjusted odds ratio (AOR) for ANC initiation was higher among those in the poorer (AOR: 1.3, 95% CI: 1.1–1.7), middle (AOR: 1.5, 95% CI: 1.2–1.9), richer (AOR: 1.4, 95% CI: 1.1–1.8), and richest (AOR: 2.7, 95% CI: 2.1–3.5) household wealth quintiles. Given the importance of appropriate ANC, it is crucial to increase awareness and coverage among women with low socioeconomic status and rural residence, among other factors studied.

## Introduction

Despite substantial reductions in maternal and childhood mortality over the past few decades, these remain high in most low- and middle-income countries (LMICs), including Bangladesh [1, 2]. The sustainable development goals aim to reduce the maternal mortality ratio (MMR) to 70 per 100,000 live births in all countries by 2030; moreover, they aim to reduce the neonatal mortality rate (NMR) to 12 per 1,000 live births by that year [3]. This will be a big challenge for LMICs like Bangladesh with high MMR and NMR. Per the latest estimates, the MMR in this country was 173 per 100,000 live births in 2017 [2], and the NMR was 30 per thousand live births in 2017–18 [4].

In LMICs, including Bangladesh, the primary causes of maternal deaths are severe bleeding, infection, high blood pressure from pre-eclampsia and eclampsia, and delivery complications [2]. On the other hand, the majority of neonatal deaths occur due to prematurity, infections, and birth asphyxia [5]. Most maternal and child deaths are preventable with currently feasible and available interventions, including antenatal care (ANC) [6–8]. The main purposes of ANC are risk identification, identification and management of pregnancy-related and concurrent health conditions, and health education [8, 9]. The goal of management is to ensure a positive pregnancy experience for the mother with fewer complications than usual before and during childbirth. ANC could also be described as the first step in the "continuum of care," where women receive information about hospital delivery, childcare, breastfeeding, birth intervals, and family planning [7, 8]. Currently, the WHO recommends initiating ANC during the first trimester of pregnancy and having at least eight contacts. However, some countries, including Bangladesh, adhere to previous guidelines regarding the number of ANC contacts/visits [8]. The Government of the People’s Republic of Bangladesh recommends having at least four ANC visits [10]. In addition to prioritizing timely initiation and an adequate number of ANC visits, the quality of ANC is crucial. Quality ANC entails having "at least four ANC visits," "at least one of these visits by a medically trained provider," and receiving basic screening and information (e.g., blood pressure measurement) [4]. According to the latest Bangladesh Demographic and Health Survey (BDHS) 2017–18, only 37% of women initiated ANC during the first trimester, 47% received at least 4 ANC visits, and only 18% received quality ANC [4]. These proportions have increased substantially over the past couple of decades. For instance, the proportions of women with at least 4 ANC visits were 6%, 11%, and 26% in 1993–94, 1999–00, and 2011, respectively. However, more than half of the women still do not receive the recommended number or quality of ANC [4, 11, 12].

Previous studies have reported disparities in the timely initiation, number, and quality of ANC in LMICs, including Bangladesh. Several individual, pregnancy-related, and socioeconomic factors impact these outcomes [13–17]. For instance, women with higher socioeconomic status (e.g., higher education level or household wealth quintile) and urban residence have a higher number of ANC visits compared to those with lower socioeconomic status or rural residence. Access to healthcare, including the distance to a health facility, can also affect the likelihood of ANC utilization [8, 17, 18]. Increasing ANC coverage is crucial to improving pregnancy outcomes and reducing maternal or neonatal deaths [8]. Identifying the factors associated with the timely initiation, number, and quality of ANC and promoting them through maternal and child health programs would be a valuable step towards enhancing ANC utilization. However, there has been limited recent epidemiological research on the disparities, distribution, and determinants of timely initiation, number, and quality of ANC coverage in Bangladesh. The primary aims of the current study are to fill these knowledge gaps by focusing on Bangladesh. The findings of this study may contribute to improving ANC coverage in Bangladesh and other similar LMICs.

## Methods

### Survey design and settings

This cross-sectional study analyzed BDHS 2017–18 data, which is a nationally representative survey conducted in Bangladesh. Bangladesh is a South Asian country with an estimated population of 170 million, and a land mass of 55,000 square miles. Approximately two-thirds of the population lives in rural areas. The majority of people practice Islam, followed by Hinduism and other religions [4]. BDHS 2017–18 collected data on major demographic, maternal, and child health indicators in Bangladesh. The survey encompassed both rural and urban regions across all divisions, which are the largest administrative units of the country. It is the eighth DHS conducted in Bangladesh as part of the worldwide DHS program. Two hundred data collectors were recruited and trained to conduct the interviews. Data collection took place between October 2017 and March 2018 [4].

First, a sample frame was prepared based on the housing and population census of 2011, consisting of a list of enumeration areas (EA). The sampling frame was designed to provide separate estimates for rural and urban regions, as well as for each administrative division. The household selection process was conducted in two stages. A total of 425 EAs were selected from rural regions, and 250 EAs were selected from urban regions. From each EA, a sample of 30 households was chosen. The survey report, methodology, sample size calculation, and questionnaires can be accessed online. The response rate for the survey was 98%, and a total of 20,127 women were interviewed from 20,160 households. For the present study, only ever-married women of reproductive age (i.e., 15-49-year-olds) with at least one pregnancy were eligible to be included, resulting in a sample size of 5,052 women [4].

### Outcomes

This study examines four outcomes related to ANC: the recommended timing of initiation (1 outcome), the number of ANC visits (2 outcomes), and the quality of ANC (1 outcome). Women were considered to have met the recommended timing of initiation if they started ANC during the first trimester of pregnancy [4]. As the Government of Bangladesh recommends at least 4 ANC visits and the WHO recommends at least 8 ANC visits, we included both as outcomes in our analysis. Lastly, the quality of ANC was defined as receiving "at least 4 ANC visits" with at least one visit conducted by a medically trained provider who incorporates the four basic ANC components, namely, body weight measurement, blood pressure measurement, urine and blood sample collection and testing, and dissemination of information about danger signs during pregnancy. Medically trained providers included qualified doctors, nurses, midwives, paramedics, family welfare visitors, community skilled birth attendants, and sub-assistant community medical officers [4].

### Exposures

Based on published studies, scientific plausibility, and the BDHS 2017–18 dataset, the following variables were selected as potential determinants of the recommended timing of initiation, number, and quality of ANC: maternal age, parity, women’s education, husband’s education, current work status, religion, household wealth status, and rural-urban place and division of residence.

Maternal age was categorized as 15–24, 25–34, and 35–49 years. Parity was categorized as ’primi’ (i.e., first pregnancy) or ’second or more’. Women were asked about the education of themselves and their husbands. Education was categorized as no formal education, primary (i.e., 1 to 5 school years), secondary (i.e., 6 to 10 school years), and college or above (i.e., 11 or more school years).

Respondents also answered questions about their work status, rural-urban place of residence, and division of residence. In 2017–18, Bangladesh had the following eight divisions: Dhaka, Chittagong, Rajshahi, Khulna, Barisal, Rangpur, Sylhet, and Mymensingh [4]. Household wealth quintiles (i.e., poorest, poorer, middle, richer, and richest) were obtained from the household wealth index score. This score was calculated by principal component analyses of basic household construction materials, water sources, sanitation facilities, electricity, and household belongings.

### Statistical analyses

First of all, the sociodemographic and socioeconomic characteristics of the respondents were described. Continuous variables were reported with the mean and standard errors (SE), while categorical variables were reported with weighted frequencies (n) and percentages (%). Next, the characteristics of the respondents were compared according to the outcomes using chi-squared tests. The prevalence of all four outcomes, along with 95% confidence intervals (CIs), was also reported.

Lastly, unadjusted and adjusted multilevel logistic regression were employed to investigate associated factors. The multilevel analysis was required to take the clustering into account as the mothers live in a cluster (i.e., geographic region) share common sociodemographic, health, and behavioral characteristics. Variables that showed associations with the outcomes in the unadjusted regression were included in the multivariable (i.e., adjusted) model. Both unadjusted odds ratios (UOR) and adjusted odds ratios (AOR), along with 95% CIs, were reported. We selected the adjusted model based on the initial selected variables and added only the variables that were significantly associated in unadjusted analyses. We also checked for the presence of multicollinearity using variance inflation factors.

The analysis was conducted using Stata 14.0 (College Station, TX, USA). The hierarchical nature of BDHS and the provided sample weights were taken into account.

## Results

A total of 5,052 women were included in the analysis (Table 1). The mean age of the women was 24.9 years (SE: 0.09), and 38.2% of them were categorized as ’primi’. Approximately 6.3% of women and 13.7% of their husbands had no formal education. More than two-thirds of the women resided in rural regions. The highest proportion of respondents, accounting for 25.6% (n = 1,293), were from Dhaka. Compared to the overall participants, the four objectives (i.e., women with ’initiation during the first trimester’, ’at least 4 visits’, ’at least 8 visits’, and ’quality ANC’) were more prevalent among women who had higher levels of education, were married to highly educated husbands, resided in urban areas, or belonged to relatively affluent household wealth quintiles. S1 to S4 Tables also provide a comparison of the study sample based on the outcomes.

**Table 1:** Sample characteristics (N = 5052).

Variable	Categories	Overall, % (n) (N = 5052)	Respondents with outcome, % (n)			
			Initiated in first trimester	At least 4 ANC	At least 8 ANC	Quality ANC
Current age (in year)	Mean (SE)	24.9 (0.1)	24.8 (0.1)	24.8 (0.1)	25.4 (0.3)	25.2 (0.2)
	15–24	53.1 (2683)	53.0 (994)	53.2 (1264)	49.5 (277)	49.2 (439)
	25–34	41.0 (2073)	42.2 (792)	41.9 (996)	45.8 (256)	46.4 (414)
	35–49	5.9 (296)	4.8 (90)	4.8 (114)	4.7 (26)	4.4 (39)
Parity	2 or more	61.8 (3121)	56.1 (1052)	56.3 (1337)	54.2 (303)	54.7 (489)
	Primi	38.2 (1931)	43.9 (824)	43.7 (1038)	45.8 (256)	45.3 (404)
Birth interval (year)	< = 2-year	10.9 (341)	8.9 (94)	9.2 (123)	7.3 (22)	8.6 (42)
	>2-year	89.1 (2780)	91.1 (958)	90.8 (1213)	92.7 (281)	91.4 (447)
Women’s education level	No education	6.3 (318)	3.4 (63)	2.6 (63)	1.4 (8)	2.0 (18)
	Primary	27.6 (1395)	19.6 (368)	20.0 (475)	17.4 (97)	13.7 (122)
	Secondary	49.0 (2475)	49.0 (919)	52.2 (1240)	48.1 (269)	49.3 (440)
	College/above	17.1 (864)	28.0 (526)	25.1 (596)	33.1 (185)	35.1 (313)
Husband’s education level	No education	13.7 (680)	8.2 (152)	8.6 (203)	7.1 (39)	6.2 (55)
	Primary	33.7 (1678)	25.0 (463)	26.6 (624)	20.5 (114)	18.2 (162)
	Secondary	34.1 (1696)	36.4 (674)	36.5 (858)	36.3 (201)	37.1 (330)
	College/above	18.5 (921)	30.4 (562)	28.2 (662)	36.1 (200)	38.5 (342)
Respondent work status	No	62.7 (3167)	68.3 (1281)	63.9 (1518)	64.7 (362)	67.9 (606)
	Yes	37.3 (1884)	31.7 (595)	36.1 (857)	35.3 (197)	32.1 (286)
Religion	Muslim	91.9 (4640)	91.3 (1713)	90.4 (2147)	90.8 (507)	90.5 (808)
	Other	8.1 (412)	8.7 (163)	9.6 (228)	9.2 (52)	9.5 (85)
Wealth quintile	Poorest	20.6 (1042)	12.3 (231)	13.5 (322)	9.3 (52)	7.7 (68)
	Poorer	20.5 (1036)	16.6 (311)	15.9 (377)	14.4 (81)	10.4 (93)
	Middle	19.2 (969)	18.1 (340)	18.6 (441)	17.8 (99)	16.8 (150)
	Richer	20.2 (1018)	20.9 (392)	22.2 (528)	20.0 (112)	24.7 (220)
	Richest	19.5 (986)	32.1 (602)	29.8 (707)	38.6 (216)	40.4 (361)
Place of residence	Urban	26.8 (1356)	34.2 (641)	33.5 (797)	40.7 (228)	40.7 (363)
	Rural	73.2 (3695)	65.8 (1235)	66.5 (1578)	59.3 (331)	59.3 (529)
Division of residence	Dhaka	25.6 (1293)	31.9 (598)	27.9 (662)	34.1 (190)	32.7 (292)
	Chittagong	21.2 (1071)	18 (337)	17.4 (413)	13.1 (73)	17.8 (159)
	Barisal	5.7 (288)	4.7 (88)	4.6 (109)	5.3 (29)	5.1 (45)
	Khulna	9.2 (464)	9.4 (176)	11.1 (265)	10.0 (56)	10.5 (94)
	Mymensingh	8.5 (431)	8.3 (156)	8.2 (196)	8.1 (45)	7.6 (67)
	Rajshahi	11.6 (587)	10.0 (188)	11.8 (281)	13.0 (73)	10.1 (90)
	Rangpur	10.6 (534)	9.7 (182)	13.3 (316)	12.7 (71)	11.6 (103)
	Sylhet	7.6 (383)	8.0 (150)	5.6 (132)	3.9 (22)	4.7 (42)

Table 2 shows the prevalence of outcome variables in the present study. More than one-third of the women initiated ANC during the first trimester, accounting for 37.1% (95% CI: 35.7–38.6). While 47.0% (95% CI: 45.5–48.5) of women received 4 or more ANC visits, only 11.1% (95% CI: 10.1–12.1) had 8 or more visits. The proportion of women who received quality ANC was 17.7% (95% CI: 16.5–18.9). The initiation of ANC during the first trimester was found to increase with women’s education, husbands’ education, household wealth, and urban residence. For instance, among women with no formal education, primary education, secondary education, and college/above education, the proportions were 19.8% (95% CI: 15.4–25.1), 26.4% (95% CI: 23.8–29.0), 37.1% (95% CI: 35.0–39.3), and 60.9% (95% CI: 57.2–64.4), respectively. Regarding the initiation of ANC during the first trimester, approximately 33.4% (95% CI: 31.7–35.2) of rural women and 47.2% (95% CI: 44.5–50.0) of urban women were observed. A similar pattern was observed for ’4 or more visits’, ’8 or more visits’, and ’quality ANC’. For example, while only 5.5% (95% CI: 3.3–9.0) of women with no formal education received quality ANC, 36.2% (95% CI: 32.8–39.8) of women with college/above education received it.

**Table 2:** Proportion of women with ANC in first trimester, 4+ visits, 8+ visits, and quality ANC.

Variable		Initiated in first trimester	At least 4 visits	At least 8 visits	Quality ANC
Current age of women (in year)	15–24	37.0 (35.0,39.1)	47.1 (45.0,49.2)	10.3 (9.1,11.7)	16.4 (14.9,18.0)
	25–34	38.2 (35.9,40.6)	48.0 (45.6,50.5)	12.3 (10.8,14.0)	20.0 (18.2,22.0)
	35–49	30.3 (24.9,36.2)	38.7 (32.8,44.9)	8.9 (6.1,12.9)	13.2 (9.6,17.8)
Parity	2 or more	33.7 (31.9,35.6)	42.8 (40.9,44.8)	9.7 (8.6,10.9)	15.7 (14.3,17.1)
	Primi	42.7 (40.2,45.2)	53.8 (51.3,56.2)	13.3 (11.7,15.0)	20.9 (19.0,23.0)
Birth interval (in year)	< = 2-year	27.5 (22.6,32.9)	36.1 (30.8,41.8)	6.5 (4.2,9.9)	12.3 (8.9,16.7)
	>2-year	34.5 (32.5,36.5)	43.6 (41.6,45.7)	10.1 (8.9,11.4)	16.1 (14.6,17.6)
Women’s education level	No education	19.8 (15.4,25.1)	19.6 (15.3,24.9)	2.5 (1.1,5.3)	5.5 (3.3,9.0)
	Primary	26.4 (23.8,29.0)	34.1 (31.4,36.9)	7.0 (5.6,8.7)	8.8 (7.3,10.6)
	Secondary	37.1 (35.0,39.3)	50.1 (47.9,52.3)	10.9 (9.6,12.3)	17.8 (16.1,19.5)
	College/above	60.9 (57.2,64.4)	69.1 (65.5,72.4)	21.4 (18.5,24.6)	36.2 (32.8,39.8)
Husband’s education level	No education	22.3 (19.1,25.9)	29.8 (26.2,33.7)	5.8 (4.1,8.0)	8.0 (6.0,10.6)
	Primary	27.6 (25.3,30.0)	37.2 (34.7,39.8)	6.8 (5.5,8.2)	9.6 (8.2,11.3)
	Secondary	39.7 (37.1,42.4)	50.6 (47.9,53.3)	11.9 (10.3,13.7)	19.4 (17.4,21.7)
	College/above	61.1 (57.5,64.5)	71.9 (68.6,75.1)	21.7 (18.9,24.8)	37.1 (33.7,40.7)
Respondent currently work	No	40.4 (38.5,42.4)	47.9 (45.9,49.9)	11.4 (10.2,12.7)	19.1 (17.6,20.7)
	Yes	31.6 (29.3,33.9)	45.5 (43.0,47.9)	10.5 (9.0,12.1)	15.2 (13.5,17.0)
Religion	Muslim	36.9 (35.4,38.5)	46.3 (44.7,47.9)	10.9 (10.0,12.0)	17.4 (16.2,18.7)
	Other	39.5 (34.4,44.8)	55.4 (50.1,60.6)	12.5 (9.4,16.6)	20.6 (16.7,25.1)
Wealth quintile	Poorest	22.2 (19.6,25.0)	30.9 (28.0,33.9)	5.0 (3.7,6.6)	6.6 (5.2,8.3)
	Poorer	30.1 (27.1,33.2)	36.4 (33.2,39.6)	7.8 (6.2,9.7)	9.0 (7.3,11.0)
	Middle	35.1 (31.7,38.6)	45.5 (42.0,49.1)	10.3 (8.3,12.6)	15.5 (13.0,18.2)
	Richer	38.5 (35.2,42.0)	51.8 (48.3,55.3)	11.0 (9.0,13.3)	21.6 (18.9,24.7)
	Richest	61.0 (57.5,64.3)	71.7 (68.5,74.7)	21.8 (19.1,24.9)	36.6 (33.3,40.0)
Place of residence	Urban	47.2 (44.5,50.0)	58.7 (56.0,61.5)	16.8 (14.8,19.0)	26.8 (24.4,29.3)
	Rural	33.4 (31.7,35.2)	42.7 (40.9,44.5)	9.0 (8.0,10.1)	14.3 (13.1,15.7)
Division of residence	Dhaka	46.3 (42.6,49.9)	51.2 (47.5,54.9)	14.7 (12.4,17.4)	22.6 (19.7,25.7)
	Chittagong	31.5 (28.3,34.8)	38.6 (35.2,42.0)	6.8 (5.3,8.8)	14.9 (12.6,17.4)
	Barisal	30.5 (26.5,34.7)	37.9 (33.7,42.4)	10.2 (7.9,13.1)	15.7 (12.6,19.3)
	Khulna	38.0 (33.7,42.4)	57.0 (52.5,61.4)	12.0 (9.5,15.0)	20.2 (16.9,23.9)
	Mymensingh	36.1 (32.2,40.3)	45.4 (41.2,49.6)	10.4 (8.2,13.2)	15.6 (12.9,18.8)
	Rajshahi	32.1 (28.1,36.4)	47.9 (43.5,52.4)	12.4 (9.7,15.7)	15.4 (12.5,18.8)
	Rangpur	34.0 (30.0,38.3)	59.2 (54.8,63.5)	13.3 (10.6,16.6)	19.3 (16.1,22.9)
	Sylhet	39.2 (35.4,43.1)	34.6 (30.9,38.4)	5.6 (4.1,7.7)	10.9 (8.8,13.5)
Overall		37.1 (35.7,38.6)	47.0 (45.5,48.5)	11.1 (10.1,12.1)	17.7 (16.5,18.9)

**Fig 1:**
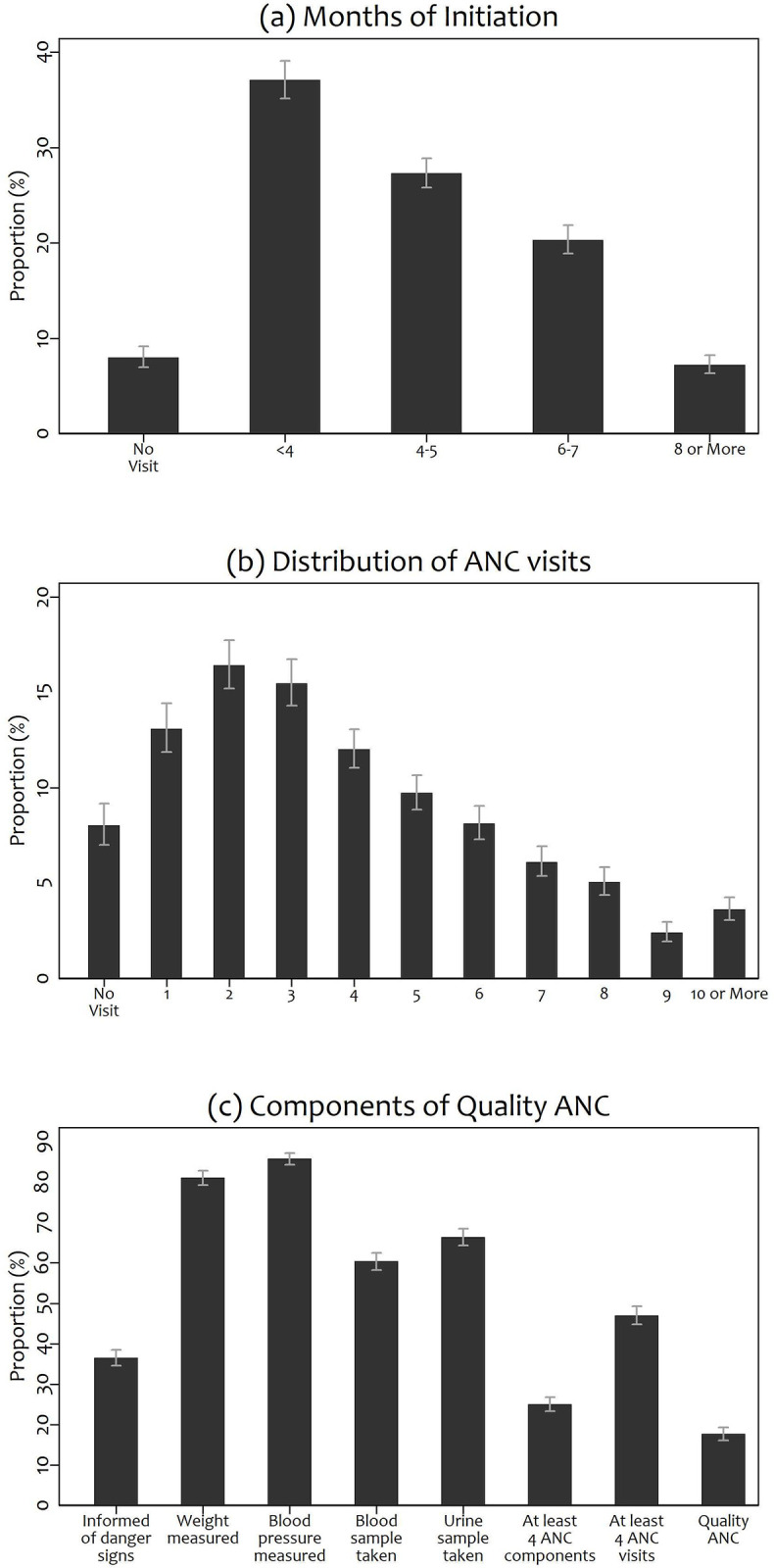
Distribution of Antenatal Care’s (a) Timing of Initiation, (b) Number of Visits, and (c) Components of Quality.

As shown in Fig 1(a), more than half (54.8%) of women initiated ANC after the first trimester. Approximately 8.0% of women did not receive any ANC, while the proportions of women with 1–3 visits and those with at least 10 visits were 45.2% and 3.6%, respectively (Fig 1(b)). Lastly, while a vast majority of women had their weight measured (81.0%) or their blood pressure recorded (85.8%), only a small proportion of women received information about danger signs (36.5%) or had at least 4 ANC components provided to them (25.0%) (Fig 1(c)).

Table 3 presents the results of multilevel logistic regression analyses. Age was found to have a significant association with the initiation of ANC in the first trimester and the quality of ANC. For example, women aged 25–34 had higher odds of ANC initiation during the first trimester (AOR: 1.2, 95% CI: 1.0–1.4) and quality ANC (AOR: 1.4, 95% CI: 1.1–1.7) compared to women aged 15–24. Higher education levels of women, husbands, and higher household wealth quintiles were associated with higher odds of all four outcomes compared to their respective counterparts. For instance, when compared to women belonging to the poorest household wealth quintile, women in the poorer (AOR: 1.3, 95% CI: 1.1–1.7), middle (AOR: 1.5, 95% CI: 1.2–1.9), richer (AOR: 1.4, 95% CI: 1.1–1.8), and richest (AOR: 2.7, 95% CI: 2.1–3.5) quintiles had higher odds of ANC initiation during the first trimester. Furthermore, rural women had lower odds of all the outcomes compared to urban women. The division of residence was also found to be significantly associated with the outcomes. The unadjusted associations are presented in the S5 Table.

**Table 3:** Adjusted odds ratio (with 95% confidence interval) for the factors associated with ANC in first trimester, 4+ visits, 8+ visits, and quality ANC.

Variable	Initiated in first trimester	At least 4 ANC visits	At least 8 ANC visits	Quality ANC visits
Current age (in year, ref: 15–24)				
25–34	1.2* (1.0,1.4)	1.2 (1.0,1.4)	1.2 (0.9,1.5)	1.4** (1.1,1.7)
35–49	1.0 (0.7,1.3)	1.0 (0.7,1.4)	1.0 (0.6,1.5)	0.9 (0.6,1.4)
Parity (ref: 2 or more)				
Primi	1.5** (1.1,2.0)	1.6** (1.2,2.1)	1.7* (1.0,2.7)	1.5 (1.0,2.2)
Birth interval (ref: < = 2-year)				
>2-year	1.3 (1.0,1.7)	1.2 (0.9,1.6)	1.4 (0.9,2.3)	1.2 (0.8,1.8)
Education level (ref: no formal education)				
Primary	1.1 (0.8,1.6)	1.8*** (1.3,2.5)	2.6* (1.2,5.6)	1.4 (0.8,2.3)
Secondary	1.5* (1.1,2.1)	2.7*** (1.9,3.9)	3.2** (1.5,6.8)	1.9* (1.1,3.3)
College/above	2.4*** (1.6,3.4)	3.4*** (2.3,5.0)	4.3*** (1.9,9.6)	2.9*** (1.6,5.2)
Husband’s education level (ref: no education)				
Primary	1.1 (0.9,1.4)	1.1 (0.9,1.4)	0.9 (0.6,1.4)	1.1 (0.7,1.5)
Secondary	1.5*** (1.2,1.9)	1.4** (1.1,1.8)	1.4 (0.9,2.1)	1.6** (1.1,2.3)
College/above	2.2*** (1.6,2.9)	2.2*** (1.6,2.9)	1.7* (1.1,2.7)	2.3*** (1.5,3.4)
Work status (ref: no)				
Yes	0.9 (0.8,1.1)	—	—	1.1 (0.9,1.3)
Religion (ref: Muslim)				
Other	—	1.3 (1.0,1.6)	—	—
Household wealth (ref: poorest)				
Poorer	1.3** (1.1,1.7)	1.1 (0.9,1.4)	1.5* (1.0,2.2)	1.2 (0.9,1.7)
Middle	1.5*** (1.2,1.9)	1.6*** (1.3,2.0)	1.8** (1.2,2.6)	1.8*** (1.3,2.5)
Richer	1.4** (1.1,1.8)	1.8*** (1.4,2.2)	1.6* (1.1,2.4)	2.3*** (1.7,3.3)
Richest	2.7*** (2.1,3.5)	2.9*** (2.1,3.8)	2.5*** (1.6,3.9)	3.5*** (2.4,5.0)
Place of residence (ref: urban)				
Rural	0.8* (0.7,1.0)	0.7*** (0.6,0.8)	0.6*** (0.5,0.8)	0.7** (0.6,0.9)
Division (ref: Dhaka)				
Chittagong	0.6*** (0.4,0.7)	0.7* (0.5,0.9)	0.5*** (0.3,0.7)	0.7 (0.5,1.0)
Barisal	0.7* (0.5,0.9)	0.8 (0.6,1.2)	1.0 (0.6,1.5)	0.9 (0.6,1.3)
Khulna	0.9 (0.7,1.2)	1.7** (1.2,2.4)	1.1 (0.7,1.6)	1.1 (0.7,1.6)
Mymensingh	1.0 (0.7,1.3)	1.4 (1.0,2.0)	1.0 (0.6,1.5)	1.2 (0.8,1.7)
Rajshahi	0.7* (0.5,1.0)	1.3 (0.9,1.8)	1.1 (0.7,1.7)	0.9 (0.6,1.3)
Rangpur	0.9 (0.7,1.3)	2.6*** (1.8,3.7)	1.3 (0.8,2.0)	1.4 (0.9,2.0)
Sylhet	1.1 (0.9,1.5)	0.7* (0.5,1.0)	0.5** (0.3,0.8)	0.7* (0.4,1.0)

*: p<0.05,

**:p<0.01,

***: p<0.001

## Discussion

After investigating the disparities in ANC initiation timing, number, and quality in Bangladesh, this study reveals that more than half of the women did not appropriately initiate, complete, or receive quality ANC. Moreover, higher socioeconomic status, such as a higher education level or household status, along with certain other sociodemographic factors like age, were found to contribute to the likelihood of these outcomes. To the best of our knowledge, this is the first nationally representative epidemiological study to comprehensively report these disparities, including the timing of initiation, number of visits, and quality of ANC, while encompassing both rural and urban regions in Bangladesh.

The association of socioeconomic status with the outcomes of this study reflects the socioeconomic disparities observed in accessing and receiving other maternal and child care services, as well as maternal and child mortality, in Bangladesh and similar LMICs [19–22]. Previous studies have reported similar findings [23–25]. Women with lower levels of education or husbands with lower levels of education may not receive adequate healthcare due to a lack of awareness or knowledge regarding the benefits of ANC during pregnancy [17, 26, 27]. Additionally, women from poorer families may face barriers to accessing ANC due to their inability to afford the associated costs [16, 17]. Higher levels of education and wealth quintiles are associated with increased odds of favorable outcomes. Education and wealth/income are interconnected, as individuals with higher education levels often earn higher incomes compared to those with lower education levels [23–25]. Although healthcare services in public hospitals in Bangladesh are available at minimal cost, the burden of travel may contribute to these disparities [4].

We also observed disparities based on place and division of residence. Disparities in access to and utilization of healthcare based on residence have been previously reported [4, 17, 19]. Furthermore, studies have indicated disparities in maternal and child health based on the location of residence. For example, the Sylhet division exhibited the highest rate of neonatal mortality, and the neonatal mortality rate in rural regions was approximately 1.5 times higher than in urban regions [4]. Since about two-thirds of the population in Bangladesh resides in rural areas, it is crucial to enhance the coverage of ANC and other healthcare services to reduce these disparities [4, 19]. Previous studies have recommended community-based interventions to raise awareness in rural regions and divisions with low utilization of maternal healthcare [8, 15, 27].

Similar to previous studies, we did not find any association between work status or religion and ANC coverage [24, 28, 29]. However, we observed an association between parity and the timing of initiation and the number of ANC visits, although it did not show any association with the quality of ANC. These findings align with multiple previous studies [17, 30, 31]. Women with a history of previous pregnancies (i.e., two or more pregnancies) may possess more knowledge about the importance of ANC. Therefore, it is crucial for future programs aimed at improving ANC coverage to consider increasing appropriate coverage among women experiencing their first pregnancy.

These findings have implications for Bangladesh and other similar LMICs. ANC serves as the initial step in the continuum of care during pregnancy, yet a majority of women did not initiate ANC during the first trimester or receive the recommended number or quality of ANC [8, 27]. Given the crucial role of ANC in reducing maternal and child mortality, future interventions should address additional barriers to accessing health services. One unique aspect of this study is that it examines the timing, number, and quality of ANC together, revealing that the factors influencing these outcomes are similar. The identified socioeconomic disparities must be taken into account. Consequently, policymakers in Bangladesh could address these identified factors collectively to enhance the outcomes under study. Moreover, while 47% of women received ANC, only 18% received quality ANC. In other words, among those who received 4 ANC visits, only one out of three received quality ANC. Additionally, only approximately 37% of participants were informed about danger signs. Without ensuring the quality of ANC, the full benefits of ANC cannot be realized. Although we did not investigate the quality of health centers where women receive ANC, they can also impact the quality of ANC provided [4].

This study possesses several notable strengths. Firstly, it had a large sample size and encompassed rural and urban regions across all administrative divisions, rendering the findings highly generalizable. Additionally, the survey achieved a high response rate and had minimal missing data, ensuring the reliability of the results. Moreover, the study employed standardized and validated instruments, definitions, and methods, enabling comparisons across women with diverse sociodemographic and socioeconomic backgrounds [4].

The present study also has certain limitations that require consideration. Firstly, we were unable to examine the association between certain factors, such as the quality of health centers. Additionally, the inclusion of pregnancy history from the past three years in this study may introduce some recall bias. It is important to note that this study is cross-sectional in nature, which limits the ability to establish causality due to the absence of temporal certainty [4].

## Conclusion

We have presented the disparities in the distributions of ANC initiation timing, number, and quality in Bangladesh. Irrespective of location or socioeconomic status, ensuring appropriate ANC coverage is crucial for enhancing overall maternal and child health in the country. The socioeconomic disparities identified in this study can guide programs and policies aimed at reducing such disparities in health outcomes. Future interventions should focus on increasing awareness regarding the significance of ANC among women with low socioeconomic status. In implementing these interventions, policymakers must also take into account various other factors, including parity and divisions of residence.

## Supporting information

S1 Table Comparison of study sample by the timing of initiation. (DOCX)

S2 Table Comparison of study sample by at least 4 ANC visits. (DOCX)

S3 Table Comparison of study sample by at least 8 ANC visits. (DOCX)

S4 Table Comparison of study sample by quality of ANC. (DOCX)

S5 Table Unadjusted odds ratio (95% confidence interval) for the association between potential factors and outcomes. (DOCX)
