# Supplementary material for: Disparities, distribution, and determinants in appropriate timely initiation, number, and quality of antenatal care in Bangladesh: Evidence from Demographic and Health Survey 2017–18
Source: PLOS Glob Public Health. 2023 Aug 23;3(8):e0002325. doi: 10.1371/journal.pgph.0002325 (PMC10446198; doi:10.1371/journal.pgph.0002325)
Supplement: S1 Table — (DOCX) [file pgph.0002325.s001.docx]

S1 Table: Comparison of study sample by the timing of initiation

| Variable | | Overall | Yes | No | p-value |
| --- | --- | --- | --- | --- | --- |
| Current age of women (in year) | 15-24 | 53.1 (2683) | 53 (994) | 53.2 (1689) | 0.067 |
|  | 25-34 | 41 (2073) | 42.2 (792) | 40.3 (1281) |  |
|  | 35-49 | 5.9 (296) | 4.8 (90) | 6.5 (206) |  |
| Parity | 2 or more | 61.8 (3121) | 56.1 (1052) | 65.2 (2069) | <0.001 |
|  | Primi | 38.2 (1931) | 43.9 (824) | 34.8 (1107) |  |
| Birth interval (in year) | <=2-year | 6.7 (341) | 5 (94) | 7.8 (247) | <0.001 |
|  | >2-year | 55 (2780) | 51.1 (958) | 57.4 (1822) |  |
|  | Primi | 38.2 (1931) | 43.9 (824) | 34.8 (1107) |  |
| Women's education level | No education | 6.3 (318) | 3.4 (63) | 8 (255) | <0.001 |
|  | Primary | 27.6 (1395) | 19.6 (368) | 32.3 (1027) |  |
|  | Secondary | 49 (2475) | 49 (919) | 49 (1556) |  |
|  | College/above | 17.1 (864) | 28 (526) | 10.6 (338) |  |
| Husband's education level | No education | 13.7 (680) | 8.2 (152) | 16.9 (528) | <0.001 |
|  | Primary | 33.7 (1678) | 25 (463) | 38.9 (1215) |  |
|  | Secondary | 34.1 (1696) | 36.4 (674) | 32.7 (1022) |  |
|  | College/above | 18.5 (921) | 30.4 (562) | 11.5 (358) |  |
| Respondent currently working | No | 62.7 (3167) | 68.3 (1281) | 59.4 (1886) | <0.001 |
|  | Yes | 37.3 (1884) | 31.7 (595) | 40.6 (1290) |  |
| Religion | Muslim | 91.9 (4640) | 91.3 (1713) | 92.2 (2927) | 0.48 |
|  | Other | 8.1 (412) | 8.7 (163) | 7.8 (249) |  |
| Wealth quintile | Poorest | 20.6 (1042) | 12.3 (231) | 25.5 (811) | <0.001 |
|  | Poorer | 20.5 (1036) | 16.6 (311) | 22.8 (725) |  |
|  | Middle | 19.2 (969) | 18.1 (340) | 19.8 (629) |  |
|  | Richer | 20.2 (1018) | 20.9 (392) | 19.7 (626) |  |
|  | Richest | 19.5 (986) | 32.1 (602) | 12.1 (385) |  |
| Place of residence | Urban | 26.8 (1356) | 34.2 (641) | 22.5 (716) | <0.001 |
|  | Rural | 73.2 (3695) | 65.8 (1235) | 77.5 (2460) |  |
| Division of residence | Dhaka | 25.6 (1293) | 31.9 (598) | 21.9 (695) | <0.001 |
|  | Chittagong | 21.2 (1071) | 18 (337) | 23.1 (734) |  |
|  | Barisal | 5.7 (288) | 4.7 (88) | 6.3 (200) |  |
|  | Khulna | 9.2 (464) | 9.4 (176) | 9.1 (288) |  |
|  | Mymensingh | 8.5 (431) | 8.3 (156) | 8.7 (275) |  |
|  | Rajshahi | 11.6 (587) | 10 (188) | 12.5 (398) |  |
|  | Rangpur | 10.6 (534) | 9.7 (182) | 11.1 (352) |  |
|  | Sylhet | 7.6 (383) | 8 (150) | 7.3 (233) |  |
