# Supplementary material for: Disparities, distribution, and determinants in appropriate timely initiation, number, and quality of antenatal care in Bangladesh: Evidence from Demographic and Health Survey 2017–18
Source: PLOS Glob Public Health. 2023 Aug 23;3(8):e0002325. doi: 10.1371/journal.pgph.0002325 (PMC10446198; doi:10.1371/journal.pgph.0002325)
Supplement: S2 Table — (DOCX) [file pgph.0002325.s002.docx]

S2 Table: Comparison of study sample by at least 4 ANC visits

| Variable | | Overall | Yes | No | p-values |
| --- | --- | --- | --- | --- | --- |
| Current age of women (in year) | 15-24 | 53.1 (2683) | 53.2 (1264) | 53 (1419) | 0.035 |
|  | 25-34 | 41 (2073) | 41.9 (996) | 40.2 (1077) |  |
|  | 35-49 | 5.9 (296) | 4.8 (114) | 6.8 (181) |  |
| Parity | 2 or more | 61.8 (3121) | 56.3 (1337) | 66.7 (1784) | <0.001 |
|  | Primi | 38.2 (1931) | 43.7 (1038) | 33.3 (893) |  |
| Birth interval (in year) | <=2-year | 6.7 (341) | 5.2 (123) | 8.1 (218) | <0.001 |
|  | >2-year | 55 (2780) | 51.1 (1213) | 58.5 (1567) |  |
|  | Primi | 38.2 (1931) | 43.7 (1038) | 33.3 (893) |  |
| Women's education level | No education | 6.3 (318) | 2.6 (63) | 9.6 (256) | <0.001 |
|  | Primary | 27.6 (1395) | 20 (475) | 34.3 (920) |  |
|  | Secondary | 49 (2475) | 52.2 (1240) | 46.1 (1234) |  |
|  | College/above | 17.1 (864) | 25.1 (596) | 10 (267) |  |
| Husband's education level | No education | 13.7 (680) | 8.6 (203) | 18.2 (477) | <0.001 |
|  | Primary | 33.7 (1678) | 26.6 (624) | 40.1 (1054) |  |
|  | Secondary | 34.1 (1696) | 36.5 (858) | 31.9 (838) |  |
|  | College/above | 18.5 (921) | 28.2 (662) | 9.8 (258) |  |
| Respondent currently working | No | 62.7 (3167) | 63.9 (1518) | 61.6 (1649) | 0.18 |
|  | Yes | 37.3 (1884) | 36.1 (857) | 38.4 (1028) |  |
| Religion | Muslim | 91.9 (4640) | 90.4 (2147) | 93.1 (2493) | 0.015 |
|  | Other | 8.1 (412) | 9.6 (228) | 6.9 (184) |  |
| Wealth quintile | Poorest | 20.6 (1042) | 13.5 (322) | 26.9 (720) | <0.001 |
|  | Poorer | 20.5 (1036) | 15.9 (377) | 24.6 (659) |  |
|  | Middle | 19.2 (969) | 18.6 (441) | 19.7 (528) |  |
|  | Richer | 20.2 (1018) | 22.2 (528) | 18.3 (491) |  |
|  | Richest | 19.5 (986) | 29.8 (707) | 10.4 (279) |  |
| Place of residence | Urban | 26.8 (1356) | 33.5 (797) | 20.9 (560) | <0.001 |
|  | Rural | 73.2 (3695) | 66.5 (1578) | 79.1 (2117) |  |
| Division of residence | Dhaka | 25.6 (1293) | 27.9 (662) | 23.6 (631) | <0.001 |
|  | Chittagong | 21.2 (1071) | 17.4 (413) | 24.6 (658) |  |
|  | Barisal | 5.7 (288) | 4.6 (109) | 6.7 (179) |  |
|  | Khulna | 9.2 (464) | 11.1 (265) | 7.5 (200) |  |
|  | Mymensingh | 8.5 (431) | 8.2 (196) | 8.8 (235) |  |
|  | Rajshahi | 11.6 (587) | 11.8 (281) | 11.4 (306) |  |
|  | Rangpur | 10.6 (534) | 13.3 (316) | 8.1 (218) |  |
|  | Sylhet | 7.6 (383) | 5.6 (132) | 9.4 (251) |  |
