# Supplementary material for: Disparities, distribution, and determinants in appropriate timely initiation, number, and quality of antenatal care in Bangladesh: Evidence from Demographic and Health Survey 2017–18
Source: PLOS Glob Public Health. 2023 Aug 23;3(8):e0002325. doi: 10.1371/journal.pgph.0002325 (PMC10446198; doi:10.1371/journal.pgph.0002325)
Supplement: S3 Table — (DOCX) [file pgph.0002325.s003.docx]

S3 Table: Comparison of study sample by at least 8 ANC visits

| Variable | | Overall | Yes | No | p-values |
| --- | --- | --- | --- | --- | --- |
| Current age of women (in year) | 15-24 | 53.1 (2683) | 49.5 (277) | 53.6 (2406) | 0.08 |
|  | 25-34 | 41 (2073) | 45.8 (256) | 40.4 (1817) |  |
|  | 35-49 | 5.9 (296) | 4.7 (26) | 6 (269) |  |
| Parity | 2 or more | 61.8 (3121) | 54.2 (303) | 62.7 (2818) | <0.001 |
|  | Primi | 38.2 (1931) | 45.8 (256) | 37.3 (1675) |  |
| Birth interval (in year) | <=2-year | 6.7 (341) | 4 (22) | 7.1 (319) | <0.001 |
|  | >2-year | 55 (2780) | 50.2 (281) | 55.6 (2499) |  |
|  | Primi | 38.2 (1931) | 45.8 (256) | 37.3 (1675) |  |
| Women's education level | No education | 6.3 (318) | 1.4 (8) | 6.9 (310) | <0.001 |
|  | Primary | 27.6 (1395) | 17.4 (97) | 28.9 (1298) |  |
|  | Secondary | 49 (2475) | 48.1 (269) | 49.1 (2206) |  |
|  | College/above | 17.1 (864) | 33.1 (185) | 15.1 (679) |  |
| Husband's education level | No education | 13.7 (680) | 7.1 (39) | 14.5 (641) | <0.001 |
|  | Primary | 33.7 (1678) | 20.5 (114) | 35.4 (1564) |  |
|  | Secondary | 34.1 (1696) | 36.3 (201) | 33.8 (1494) |  |
|  | College/above | 18.5 (921) | 36.1 (200) | 16.3 (721) |  |
| Respondent currently working | No | 62.7 (3167) | 64.7 (362) | 62.4 (2805) | 0.36 |
|  | Yes | 37.3 (1884) | 35.3 (197) | 37.6 (1687) |  |
| Religion | Muslim | 91.9 (4640) | 90.8 (507) | 92 (4132) | 0.34 |
|  | Other | 8.1 (412) | 9.2 (52) | 8 (360) |  |
| Wealth quintile | Poorest | 20.6 (1042) | 9.3 (52) | 22 (990) | <0.001 |
|  | Poorer | 20.5 (1036) | 14.4 (81) | 21.3 (955) |  |
|  | Middle | 19.2 (969) | 17.8 (99) | 19.4 (870) |  |
|  | Richer | 20.2 (1018) | 20 (112) | 20.2 (907) |  |
|  | Richest | 19.5 (986) | 38.6 (216) | 17.2 (771) |  |
| Place of residence | Urban | 26.8 (1356) | 40.7 (228) | 25.1 (1129) | <0.001 |
|  | Rural | 73.2 (3695) | 59.3 (331) | 74.9 (3364) |  |
| Division of residence | Dhaka | 25.6 (1293) | 34.1 (190) | 24.5 (1103) | <0.001 |
|  | Chittagong | 21.2 (1071) | 13.1 (73) | 22.2 (998) |  |
|  | Barisal | 5.7 (288) | 5.3 (29) | 5.8 (259) |  |
|  | Khulna | 9.2 (464) | 10 (56) | 9.1 (409) |  |
|  | Mymensingh | 8.5 (431) | 8.1 (45) | 8.6 (386) |  |
|  | Rajshahi | 11.6 (587) | 13 (73) | 11.4 (514) |  |
|  | Rangpur | 10.6 (534) | 12.7 (71) | 10.3 (463) |  |
|  | Sylhet | 7.6 (383) | 3.9 (22) | 8 (362) |  |
