# Supplementary material for: Disparities, distribution, and determinants in appropriate timely initiation, number, and quality of antenatal care in Bangladesh: Evidence from Demographic and Health Survey 2017–18
Source: PLOS Glob Public Health. 2023 Aug 23;3(8):e0002325. doi: 10.1371/journal.pgph.0002325 (PMC10446198; doi:10.1371/journal.pgph.0002325)
Supplement: S4 Table — (DOCX) [file pgph.0002325.s004.docx]

S4 Table: Comparison of study sample by quality of ANC

| Variable | | Overall | Yes | No | p-values |
| --- | --- | --- | --- | --- | --- |
| Current age of women (in year) | 15-24 | 53.1 (2683) | 49.2 (439) | 54 (2244) | 0.001 |
|  | 25-34 | 41 (2073) | 46.4 (414) | 39.9 (1658) |  |
|  | 35-49 | 5.9 (296) | 4.4 (39) | 6.2 (257) |  |
| Parity | 2 or more | 61.8 (3121) | 54.7 (489) | 63.3 (2632) | <0.001 |
|  | Primi | 38.2 (1931) | 45.3 (404) | 36.7 (1527) |  |
| Birth interval (in year) | <=2-year | 6.7 (341) | 4.7 (42) | 7.2 (299) | <0.001 |
|  | >2-year | 55 (2780) | 50.1 (447) | 56.1 (2333) |  |
|  | Primi | 38.2 (1931) | 45.3 (404) | 36.7 (1527) |  |
| Women's education level | No education | 6.3 (318) | 2 (18) | 7.2 (301) | <0.001 |
|  | Primary | 27.6 (1395) | 13.7 (122) | 30.6 (1273) |  |
|  | Secondary | 49 (2475) | 49.3 (440) | 48.9 (2035) |  |
|  | College/above | 17.1 (864) | 35.1 (313) | 13.2 (551) |  |
| Husband's education level | No education | 13.7 (680) | 6.2 (55) | 15.3 (625) | <0.001 |
|  | Primary | 33.7 (1678) | 18.2 (162) | 37.1 (1516) |  |
|  | Secondary | 34.1 (1696) | 37.1 (330) | 33.4 (1366) |  |
|  | College/above | 18.5 (921) | 38.5 (342) | 14.2 (579) |  |
| Respondent currently working | No | 62.7 (3167) | 67.9 (606) | 61.6 (2561) | 0.003 |
|  | Yes | 37.3 (1884) | 32.1 (286) | 38.4 (1598) |  |
| Religion | Muslim | 91.9 (4640) | 90.5 (808) | 92.1 (3832) | 0.26 |
|  | Other | 8.1 (412) | 9.5 (85) | 7.9 (327) |  |
| Wealth quintile | Poorest | 20.6 (1042) | 7.7 (68) | 23.4 (973) | <0.001 |
|  | Poorer | 20.5 (1036) | 10.4 (93) | 22.7 (943) |  |
|  | Middle | 19.2 (969) | 16.8 (150) | 19.7 (820) |  |
|  | Richer | 20.2 (1018) | 24.7 (220) | 19.2 (798) |  |
|  | Richest | 19.5 (986) | 40.4 (361) | 15 (626) |  |
| Place of residence | Urban | 26.8 (1356) | 40.7 (363) | 23.9 (993) | <0.001 |
|  | Rural | 73.2 (3695) | 59.3 (529) | 76.1 (3166) |  |
| Division of residence | Dhaka | 25.6 (1293) | 32.7 (292) | 24.1 (1001) | <0.001 |
|  | Chittagong | 21.2 (1071) | 17.8 (159) | 21.9 (912) |  |
|  | Barisal | 5.7 (288) | 5.1 (45) | 5.8 (243) |  |
|  | Khulna | 9.2 (464) | 10.5 (94) | 8.9 (371) |  |
|  | Mymensingh | 8.5 (431) | 7.6 (67) | 8.7 (364) |  |
|  | Rajshahi | 11.6 (587) | 10.1 (90) | 11.9 (497) |  |
|  | Rangpur | 10.6 (534) | 11.6 (103) | 10.4 (431) |  |
|  | Sylhet | 7.6 (383) | 4.7 (42) | 8.2 (341) |  |
