# Supplementary material for: Disparities, distribution, and determinants in appropriate timely initiation, number, and quality of antenatal care in Bangladesh: Evidence from Demographic and Health Survey 2017–18
Source: PLOS Glob Public Health. 2023 Aug 23;3(8):e0002325. doi: 10.1371/journal.pgph.0002325 (PMC10446198; doi:10.1371/journal.pgph.0002325)
Supplement: S5 Table — (DOCX) [file pgph.0002325.s005.docx]

S5 Table: Unadjusted odds ratio (95% confidence interval) for the association between potential factors and outcomes

| Variable | Initiated during first trimester | At least 4 ANC visits | At least 8 ANC visits | Quality ANC visits |
| --- | --- | --- | --- | --- |
| Current age (in year, ref: 15-24) | Ref. (1.0) | Ref. (1.0) | Ref. (1.0) | Ref. (1.0) |
| 25-34 | 1.0 (0.9,1.2) | 1.0 (0.9,1.1) | 1.0 (0.9,1.3) | 1.3** (1.1,1.5) |
| 35-49 | 0.8 (0.6,1.0) | 0.7** (0.5,0.9) | 0.8 (0.5,1.2) | 0.8 (0.5,1.1) |
| Parity (ref: 2 or more) | Ref. (1.0) | Ref. (1.0) | Ref. (1.0) | Ref. (1.0) |
| Primi | 1.5*** (1.3,1.6) | 1.6*** (1.4,1.8) | 1.5*** (1.2,1.8) | 1.4*** (1.2,1.7) |
| Birth interval (ref: <=2-year) | Ref. (1.0) | Ref. (1.0) | Ref. (1.0) | Ref. (1.0) |
| >2-year | 1.3* (1.0,1.7) | 1.4* (1.0,1.8) | 1.7* (1.1,2.7) | 1.4 (1.0,2.0) |
| Primi | 1.9*** (1.4,2.4) | 2.1*** (1.6,2.7) | 2.4*** (1.5,3.8) | 1.9*** (1.3,2.8) |
| Education level (ref: no education) | Ref. (1.0) | Ref. (1.0) | Ref. (1.0) | Ref. (1.0) |
| Primary | 1.3 (1.0,1.8) | 2.1*** (1.5,2.9) | 3.0** (1.4,6.3) | 1.6 (0.9,2.7) |
| Secondary | 2.2*** (1.6,3.0) | 4.4*** (3.2,6.2) | 5.2*** (2.5,10.8) | 3.5*** (2.1,5.8) |
| College/above | 6.1*** (4.4,8.4) | 10.1*** (7.1,14.5) | 11.5*** (5.4,24.3) | 10.3*** (6.1,17.5) |
| Husband's education level (ref: no education) | Ref. (1.0) | Ref. (1.0) | Ref. (1.0) | Ref. (1.0) |
| Primary | 1.2 (1.0,1.6) | 1.4** (1.1,1.7) | 1.2 (0.8,1.8) | 1.3 (0.9,1.9) |
| Secondary | 2.2*** (1.8,2.8) | 2.5*** (2.0,3.1) | 2.3*** (1.6,3.4) | 3.0*** (2.1,4.1) |
| College/above | 5.4*** (4.2,6.8) | 6.0*** (4.7,7.8) | 4.6*** (3.1,6.8) | 7.5*** (5.4,10.6) |
| Work status (ref: no) | Ref. (1.0) | Ref. (1.0) | Ref. (1.0) | Ref. (1.0) |
| Yes | 0.7*** (0.6,0.8) | 0.9 (0.8,1.1) | 0.9 (0.7,1.1) | 0.8* (0.7,1.0) |
| Religion (ref: Muslim) | Ref. (1.0) | Ref. (1.0) | Ref. (1.0) | Ref. (1.0) |
| Other | 1.0 (0.8,1.3) | 1.3* (1.0,1.7) | 1.2 (0.8,1.7) | 1.2 (0.9,1.7) |
| Household wealth quintile (ref: poorest) | Ref. (1.0) | Ref. (1.0) | Ref. (1.0) | Ref. (1.0) |
| Poorer | 1.5*** (1.2,1.9) | 1.2* (1.0,1.5) | 1.7** (1.1,2.4) | 1.4* (1.0,1.9) |
| Middle | 2.0*** (1.6,2.5) | 2.1*** (1.7,2.6) | 2.4*** (1.7,3.5) | 2.6*** (1.9,3.5) |
| Richer | 2.3*** (1.9,2.9) | 2.7*** (2.2,3.4) | 2.7*** (1.9,3.9) | 3.9*** (2.9,5.3) |
| Richest | 6.0*** (4.9,7.5) | 5.9*** (4.6,7.4) | 5.6*** (3.9,8.0) | 8.4*** (6.2,11.4) |
| Place of residence (ref: urban) | Ref. (1.0) | Ref. (1.0) | Ref. (1.0) | Ref. (1.0) |
| Rural | 0.5*** (0.4,0.6) | 0.5*** (0.4,0.6) | 0.4*** (0.3,0.5) | 0.4*** (0.3,0.5) |
| Division of residence (ref: Dhaka) | Ref. (1.0) | Ref. (1.0) | Ref. (1.0) | Ref. (1.0) |
| Chittagong | 0.5*** (0.4,0.7) | 0.6** (0.4,0.8) | 0.4*** (0.2,0.6) | 0.6** (0.4,0.9) |
| Barisal | 0.5*** (0.4,0.7) | 0.6** (0.4,0.8) | 0.7 (0.4,1.1) | 0.6** (0.4,0.8) |
| Khulna | 0.7 (0.5,1.0) | 1.4 (1.0,2.0) | 0.9 (0.6,1.4) | 0.9 (0.6,1.3) |
| Mymensingh | 0.6** (0.5,0.9) | 0.8 (0.6,1.2) | 0.6* (0.4,1.0) | 0.6* (0.4,0.9) |
| Rajshahi | 0.6*** (0.4,0.8) | 0.9 (0.6,1.3) | 0.8 (0.5,1.3) | 0.6* (0.4,1.0) |
| Rangpur | 0.7* (0.5,0.9) | 1.6* (1.1,2.3) | 0.8 (0.5,1.3) | 0.8 (0.6,1.3) |
| Sylhet | 0.8 (0.6,1.1) | 0.5*** (0.3,0.7) | 0.3*** (0.2,0.5) | 0.4*** (0.3,0.6) |
